# Pediatric HyperCKemia: a 13-year retrospective study and predictors of neuromuscular disease and metabolic myopathy

**DOI:** 10.1007/s00431-026-06914-6

**Published:** 2026-04-10

**Authors:** Inês Aires Martins, Joana Baptista de Lima, Margarida Paiva Coelho, Anabela Bandeira, Esmeralda Martins, Joana Correia

**Affiliations:** 1 Pediatrics Department, Centro Materno-Infantil Do Norte Albino Aroso, Unidade Local de Saúde de Santo António, Porto, Portugal; 2 Reference Center of Inherited Metabolic Diseases, Unidade Local de Saúde de Santo António, Porto, Portugal

**Keywords:** Creatine Kinase, Rhabdomyolysis, Neuromuscular Diseases, Metabolic Diseases

## Abstract

HyperCKemia is a frequent laboratory finding in pediatric practice and may reflect a wide spectrum of conditions, ranging from benign transient states to rare primary neuromuscular disorders. The lack of pediatric-specific diagnostic algorithms complicates risk stratification and clinical decision-making. A retrospective cohort study was conducted, including patients under 18 years of age with hyperCKemia referred to a tertiary reference center between January 2017 and December 2024. Clinical, laboratory, genetic, and histological data were collected. Statistical analyses included univariate testing and logistic regression to identify predictors of primary disease. A total of 119 patients were included (median age 10 years; 76.5% male). Most were symptomatic at presentation (87.4%), and 80.3% met criteria for rhabdomyolysis. A primary disorder was identified in 25.2% of patients, while 52.1% had secondary causes, predominantly viral myositis, and 22.7% remained undiagnosed. Metabolic myopathies accounted for the majority of primary diagnoses (60.0%), followed by muscular dystrophies (23.3%) and inflammatory myopathies (13.3%). Persistent hyperCKemia and male sex emerged as strong independent predictors of primary disease (*OR* 8.5 and 5.1, respectively). Muscle weakness and exercise intolerance were exclusive to primary disorders. Genetic testing yielded a diagnosis in 26.8% of tested patients, with targeted next-generation sequencing proving particularly valuable.

*Conclusions:* In pediatric hyperCKemia, persistent CK elevation and male sex are key predictors of underlying primary neuromuscular disease. A phenotype-driven, stepwise diagnostic approach incorporating early genetic testing may improve diagnostic yield and optimize resource utilization.

**Table:** 

**What is Known:** • *HyperCKemia in children is most frequently secondary to benign conditions such as viral myositis, while primary neuromuscular disorders are less common but clinically significant.* • *Creatine kinase levels lack specificity, and the diagnostic approach increasingly relies on genetic testing.*
**What is New:** • *Persistent hyperCKemia and male sex were identified as independent predictors of primary disease.* • *Most primary disorders occurred in patients with CK over 1000 UI/L, and higher inter-crisis CK levels were associated with an increased likelihood of underlying disease.*

**Supplementary Information:**

The online version contains supplementary material available at 10.1007/s00431-026-06914-6.

## Background

### Creatine kinase and Rhabdomyolysis

Creatine kinase (CK) is an enzyme found in skeletal muscle, myocardium, and brain where it catalyzes the reaction of creatine phosphate and adenosine diphosphate to creatine and adenosine triphosphate, producing energy(1–4). The upper limit of normal for CK varies based on the laboratory test results, gender, age, and race (2,3). When muscle is stressed or inflamed, the sarcoplasmic membrane becomes permeable and leaks cytosolic enzymes like CK into the bloodstream. CK values may become elevated within 12 h of injury, peak at 24 to 72 h, and return to normal in roughly 5 days, depending on the degree of injury and appropriate therapy (1,3). Rhabdomyolysis presents as a triad of myalgia, weakness and myoglobinuria, which manifests as the classically described tea-colored urine. However, this triad is only observed in less than 10% of patients and, despite this cluster of findings, there is no consensual definition for rhabdomyolysis. Most authors consider rhabdomyolysis when serum CK levels are greater than 5 times the upper limit of normal or higher than 1000 U/L, although clinical presentations can vary greatly (5–7). The most important complications of rhabdomyolysis are acute kidney injury (AKI), cardiac arrhythmia, disseminated intravascular coagulation and compartment syndrome. It should be noted that the risk of AKI is generally very low with CK levels under 15 000–20 000 U/L (3). Nevertheless, even in the presence of an asymptomatic hyperCKemia, patients may have an unknown genetic predisposition to develop malignant hyperthermia, typically due to mutations in the RYR1 gene (3).

### HyperCKemia etiology

HyperCKemia doesn’t always indicate a primary muscle disorder, and some patients may even have a benign, idiopathic hyperCKemia (Table 1) (1–3,8). A study including 260 children with asymptomatic hyperCKemia revealed that just a minority (5%) was diagnosed with a primary disorder within the subsequent 2 years, most commonly associated with a pattern of persistently elevated CK level, although this pattern did not significantly discriminate between primary and secondary disorders (9).

**Table 1:** Non-neuromuscular and neuromuscular causes of hyperCKemia

Secondary Causes	Primary Causes
Strenuous muscle exercise (mainly eccentric)	**Muscular dystrophies**
Viral illness	Duchenne and Becker muscular dystrophies
Trauma	Dystrophin mutations in female carriers
Surgery	Limb girdle muscular dystrophy
Medications (statins, fibrates, colchicine, zidovudine, isotretinoin, clozapine, hydroxychloroquine)	Congenital muscular dystrophies
Toxins (alcohol, heroin, cocaine)	Myofibrillar myopathy
Endocrine (hypothyroidism, hyperparathyroidism, Cushing syndrome, acromegalia)	Desmin-related myofibrillar myopathy
Metabolic (hypokalemia, hyponatremia, hypophosphatemia)	Myotonic dystrophy
Neuroacanthocytosis syndromes	**Metabolic myopathies**
Malignancy	Disorders of glycogenolysis/glycolysis
Chronic cardiac disease	Disorders of lipid metabolism
	Mitochondrial myopathies
	**Inflammatory myopathies**
	Juvenile dermatomyositis
	Inclusion body myositis
	Hypomyopathic dermatomyositis
	Sarcoid myopathy
	**Others**
	Malignant hyperthermia syndrome
	Motor neuron diseases
	Charcot-Marie-Tooth disease

#### Secondary causes

These are secondary causes of hyperCKemia and are typically associated with mild elevations in CK levels. The most common cause is recent viral infection, particularly with agents such as influenza or enteroviruses, which may lead to transient myositis (2,3). Post-viral CK elevations are usually self-limited and not indicative of an underlying neuromuscular disorder, particularly in the pediatric population. Other contributing factors include vigorous physical activity and certain medications (3,4).

#### Primary causes

##### Muscular dystrophies

Muscular dystrophies should always be considered in individuals with persistent hyperCKemia and clinical signs of myopathy such as progressive muscle weakness, exercise intolerance, calf hypertrophy, or delayed motor milestones. Dystrophinopathies are the most common muscular dystrophies (2).

##### Metabolic myopathies

This encompasses a group of rare and diverse disorders caused by defects in glycogenolysis/glycolysis, fatty acid transport and oxidation, and mitochondrial respiratory chain. Disorders of glycogenolysis/glycolysis cause muscle contracture and exertional fatigue with moderate activity; high-intensity exercise may lead to rhabdomyolysis. Disorders of lipid metabolism can cause either chronic symptoms such as weakness and exercise intolerance with histology showing intramuscular lipid accumulation, or episodic rhabdomyolysis triggered by fasting, fever, or prolonged exercise. Most have normal neurologic examination and normal CK between acute episodes (2,10). Mitochondrial myopathies have heterogeneous clinical manifestations, being weakness a common feature. HyperCKemia is not typically the predominant finding, especially in pediatric patients.

##### Inflammatory nyopathies

These should be considered in the differential diagnosis of hyperCKemia if there is subacute onset of muscle weakness (predominantly proximal symmetric) associated with rash, myalgia and/or arthralgia/arthritis. Idiopathic inflammatory myopathies rarely present with asymptomatic hyperCKemia (1,11). Juvenile dermatomyositis is the most common type of inflammatory myopathy in children (2).

### Approach of HyperCKemia

The European Federation of Neurological Societies (EFNS) guidelines and a recent review article recommend conducting further investigation in patients younger than 25 years with apparently idiopathic hyperCKemia if CK levels are found higher than the upper limit of normal in 3 different occasions regardless of the presence of symptoms (8,12). In symptomatic cases a precise diagnosis can be established in up to 75% of cases using the diagnostic resources available today (3). Next-generation sequencing (NGS) has recently been proposed as a cost-effective first tier strategy for the molecular diagnosis of inherited neuromuscular disorders (13,14). RNA sequencing from the muscle tissue of patients with undiagnosed muscular dystrophy is likely to provide a significantly higher diagnostic yield than NGS, though is not yet as widely available (14).

Most of the studies evaluating the yield of diagnostic testing in patients with hyperCKemia have focused on adults and were conducted prior to the routine and early use of NGS tests, with patients frequently undergoing invasive diagnostic testing. Additionally, neuromuscular conditions affecting the pediatric population are substantially different.

Despite muscle biopsy remaining an important part of the diagnostic process, it is currently accepted that it should be reserved as a second-tier exam (13,14) considering that nonspecific myopathic changes are frequent (16–83%). A muscle biopsy including specific stains for sarcolemmal proteins for muscular dystrophy and biochemical muscle enzyme analysis for metabolic myopathies is diagnostic in only 20% to 25% cases of asymptomatic elevated CK on average (8,13). Previous studies indicate that a definitive diagnosis through muscle biopsy is often not achieved: in three large studies comprising 323 patients, specific diagnoses were made in only 28%, while nonspecific myopathic changes were found in 43% and normal biopsies in 29% (8).

### Aim

Considering the lack of studies of diagnostic approach of hyperCKemia in pediatric population, we performed a single center retrospective study, aiming to fill the gap in this age group and to identify predictive factors for the diagnosis of neuromuscular diseases and metabolic myopathies.

## Methods

### Study design and sample

A cross-sectional, descriptive and analytical cohort study was conducted including patients under 18 years-old with hyperCKemia, referred to the Reference Center of Inherited Metabolic Disorders of a tertiary Portuguese hospital between January 2017 and December 2024. Individuals with hyperCKemia and a previously known myopathy diagnosis were excluded from the study, including those referred from the newborn screening.

### Data collection and variable definition

All data was extracted from electronic medical records, anonymized and curated in a local and restricted-access database. Demographic, clinical and laboratorial variables were collected, including age, sex, medical history, clinical manifestations, investigation approach and final diagnosis.

HyperCKemia was considered for values above 1.5 times the upper limit of normal, with a reference range of [24–173] IU/L, according to EFNS guidelines (8). All CK levels were analyzed in the same laboratory. CK levels greater than 5 times the upper limit of normal or higher than 1000 IU/L, in combination with acute muscle symptoms such as myalgia, muscle weakness and exercise intolerance, were used as the diagnostic criteria for rhabdomyolysis (5–7). Urine myoglobin was not routinely available and myoglobinuria was inferred from patient-reported dark or tea-colored urine or a false-positive hematuria.

Persistent hyperCKemia was defined as elevated CK levels on at least two occasions over a period of more than 8 weeks, whereas non-persistent hyperCKemia refers to transient or isolated elevations (8).

AKI was defined according to the KDIGO (Kidney Disease: Improving Global Outcome) criteria based on either an absolute rise in serum creatinine (SCr) level or a change in urine output (15). When baseline serum creatinine level was unknown, the upper limit of normal for the laboratory reference value was used.

Genetic testing was performed either through single-gene testing, when a specific disorder was suspected based on clinical evaluation, or through NGS panels covering multiple genes associated with rhabdomyolysis and hereditary myopathies (full panel list available in Supplementary Table 1).

Patients were classified in following categories based on last known status: primary cause, secondary cause, or indeterminate. Primary diagnoses were further characterized as muscular dystrophy, metabolic myopathy, inflammatory myopathy or other. Patients with muscular dystrophy were further subclassified by type. Ambiguous classification was resolved by consensus of the authors. A definitive diagnosis was based upon genetic confirmation following ACMG (American College of Medical Genetics and Genomics) guidelines (16).

### Ethics

This research complies with all national regulations and institutional policies and is in accordance with the tenets of the Helsinki Declaration. The study was approved by the center’s Ethics Committee. Data were anonymized without clinical data that could allow patient identification.

### Statistical analysis

Statistical analysis was performed using IBM® SPSS® Statistics 28.0. Categorical variables were expressed as frequencies (n) and percentages (%), and continuous variables as means (M) and standard deviations (SD) or medians (Mdn) and interquartile ranges (IQR) for variables with skewed distributions. Normality was assessed using the Shapiro–Wilk test or skewness and kurtosis, as appropriate. Independent samples t-tests were used to compare quantitative variables after testing for homogeneity of variance using Levene’s test. If the normality assumption was not met, the Mann–Whitney U test was used. Odds ratios (OR) and 95% confidence intervals (CI) were calculated. Differences in the distribution of categorical variables were assessed by Chi-square and Fisher exact tests. A logistic regression analysis was conducted to identify predictive factors for the diagnosis of neuromuscular diseases and metabolic myopathies. All *p* values were two-sided and were considered statistically significant if < 0.05.

## Results

A total of 119 individuals were included, with a median age of 10 years (IQR = 11) and male predominance (*n* = 91; 76.5%).

### Clinical presentation

Most patients (n = 104; 87.4%) were symptomatic at the time of the referral, primarily presenting with clinical suspicion of myositis (n = 66; 63.5%), myalgia (n = 23; 22.1%), muscle weakness (n = 15; 14.4%) and exercise intolerance (n = 16; 15.4%). Neurological symptoms such as dystonia and spasticity were observed in 2 cases (1.8%). The remaining 15 patients (12.6%) were asymptomatic, with hypercKemia found incidentally during routine analytical evaluation.

### Laboratorial presentation

The median peak CK value was 4921 IU/L (IQR = 11,442) and median inter-crisis value was 133 IU/L (IQR = 187). Persistent CK elevation was observed in 32 patients (26.9%), and 30 patients (25.2%) experienced recurrent episodes. Rhabdomyolysis was diagnosed in 94 patients (80.3%), accompanied by macroscopic hematuria in 28 cases (23.5%) and by stage 1 acute kidney injury in 5 (4.2%).

### Diagnosis

After complete diagnostic work-up, 30 patients (25.2%) were found to have a primary disorder, 62 (52.1%) had a secondary cause for hyperCKemia, and the remaining 27 (22.7%) are still undergoing investigation.

**Fig. 1:**
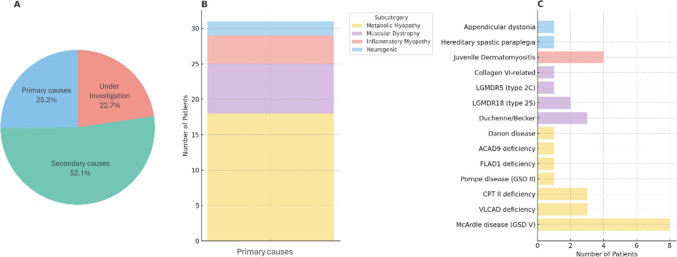
**A** diagnostic categories. **B.** Distribution of primary disorders by subtype category. **C.** Detailed breakdown of primary disorders by subtype category

Among the primary disorders identified, the majority were metabolic myopathies (n = 18; 60.0%): 9 disorders of glycogenolysis/glycolysis (8 glycogen storage disease type V (McArdle disease) and 1 glycogen storage disease type II (Pompe disease), 7 disorders of lipid metabolism (3 very long-chain acyl-CoA dehydrogenase (VLCAD) deficiency, 3 carnitine palmitoyltransferase II (CPT II) deficiency and 1 FLAD1-related FAD synthase deficiency), 2 mitochondrial myopathy (1 ACAD9 deficiency-associated mitochondrial complex I deficiency and 1 Danon disease). A muscular dystrophy was identified in 7 (23.3%) of the primary cases, including 3 limb-girdle muscular dystrophies (2 type 2S/LGMDR18 and 1 type 2 C/LGMDR5), 3 Duchenne/Becker muscular dystrophies, and 1 collagen VI-related muscular dystrophy. An inflammatory myopathy was the cause in 4 (13.3%) subjects, all of which were juvenile dermatomyositis. Neurogenic neuromuscular disorders included 1 case of hereditary spastic paraplegia and 1 movement disorder (appendicular dystonia). Of note, one patient was diagnosed with both McArdle disease and LGMDR18. Final diagnoses are represented in Fig. 1.

Secondary causes were associated with an acute viral infection (n = 50; 80.6%), with influenza B and influenza A being the most identified agents (n = 16; 32.0% and n = 7; 14.0%, respectively), as well as intense physical exercise (n = 12; 19.4%).

**Fig. 2:**
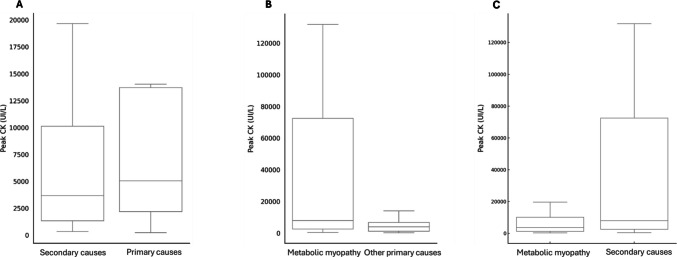
**A** comparison of peak CK value between secondary and primary disorders (p = 0.171). **B.** Comparison of peak CK value between metabolic myopathies and other primary disorders (p = 0.040). **C.** Comparison of peak CK value between metabolic myopathies and secondary disorders (p = 0.046)

**Fig. 3:**
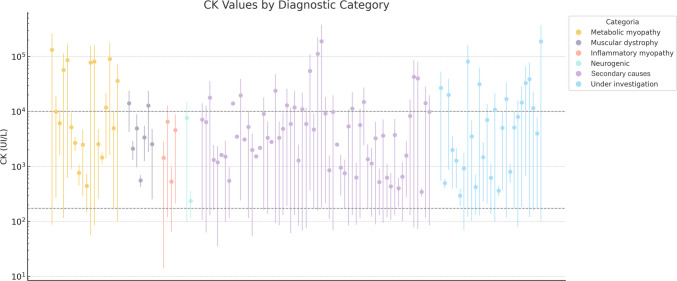
Individual CK peak and minimum values across diagnostic categories

Median peak CK value for patients with a primary disorder was higher (5063 UI/L, IQR = 17,581 *vs.* 4681 UI/L, IQR = 11,732) but it did not reach statistical significance (p = 0.171, CI −3674–20,410) (Fig. 2A). On the other hand, the minimum inter crisis CK level in those with a primary disorder was significantly higher (277.5 UI/L, IQR = 1155 *vs.* 128.5 UI/L, IQR = 93; CI 124–535, p = 0.002). Coincidentally, subjects with a primary disorder were eight times more likely to have persistently elevated CK levels (OR = 8.0, CI 3–20, p < 0.001), which was observed in over half the patients (n = 18, 60.0%) compared to 15.7% (n = 14) without. Recurrence of hyperCKemia was similar in both groups (20.0% in primary *vs* 27.0% in secondary, p = 0.447). Of note, a similar pattern was noted when comparing metabolic myopathies to other neuromuscular disorders. Patients with a metabolic myopathy presented with significantly higher median peak CK (7984 UI/L, IQR = 75,980 *vs.* 3674 UI/L, IQR = 8507; CI −44,273—−1114, p = 0.040) (Fig. 2B). Peak CK in metabolic myopathies was also significantly higher than in secondary disorders (CI 480–46103, p = 0.046) (Fig. 2C). Maximum and minimum CK values for each patient among the different diagnostic categories are represented in Fig. 3.

Most primary disorders were identified in patients with peak CK > 1000 UI/L (25 out of 30 cases, 83.3%), and of the patients with peak CK less than 5 times the upper limit of normal and no muscle weakness (n = 14, 11.8%), only 1 had a primary disorder—Pompe disease (CK 763 UI/L). Likewise, both weakness and intolerance to physical activity were significantly more common in primary conditions (p < 0.001, CI 0.1–0.2). Neither of these manifestations were found in any subject without a neuromuscular disorder. Patients who were ultimately diagnosed with a primary disorder had documented weakness at presentation in 14 (46.7%) cases and physical exercise intolerance in 15 (50.0%). Second-wind phenomenon was present in 5 of the 8 subjects with McArdle disease. Secondary cases with hyperCKemia, when symptomatic, presented mostly with myalgia.

Of the 27 patients (22.7%) with hyperCKemia who do not yet have a diagnosis, 20 (74.1%) had a peak CK at presentation > 1000 UI/L and 9 (33.3%) have persistent CK elevation.

Detailed descriptive analysis of the sample according to the final diagnosis is represented in Table 2.

**Table 2:** Descriptive analysis of the sample according to the final diagnosis. Categorical variables are expressed as frequencies and percentages, and continuous variables as median and interquartile ranges

	Primary Causes				Secondary Causes	Under investigation	*p-value* (Primary *vs* Secondary)
	Metabolic myopathy	Muscular dystrophy	Inflammatory myopathy	Neurogenic			
	*n* = 18	*n* = 7	*n* = 4	*n* = 2	*n* = 62	*n* = 27	
Sex (male)	9 (50.0)	4 (57.1)	2 (50.0)	1 (50.0)	48 (77.4)	27 (100.0)	**0.019**
Age (years)	14 (11)	4 (6)	5 (8)	4 (-)	10 (10)	15 (9)	0.587
Muscle weakness	4 (22.2)	6 (85.7)	3 (75.0)	2 (100.0)	0 (0.0)	0 (0.0)	** < 0.001**
Exercise intolerance	12 (66.7)	2 (28.6)	1 (25.0)	0 (0.0)	0 (0.0)	0 (0.0	** < 0.001**
Peak CK (UI/L)	7984 (75,980)	3356 (10,638)	2999 (5262)	3911 (-)	3674 (188,527)	5089 (19,082)	0.171
Persistent hyperCKemia	11 (61.1%)	7 (100.0%)	1 (25.0%)	0 (0.0%)	5 (8.1%)	9 (33.3%)	** < 0.001**
Recurrent hyperCKemia	6 (33.3%)	0 (0.0%)	0 (0.0%)	0 (0.0%)	17 (27.4%)	7 (25.9%)	0.447
Rhabdomyolysis	15 (83.3%)	6 (85.7%)	3 (75.0%)	1 (50.0%)	49 (79.0%)	21 (77.8%)	0.783
Macroscopic hematuria	7 (38.9%)	3 (42.9%)	1 (25.0%)	0 (0.0%)	8 (12.9%)	9 (33.3%)	**0.008**
Kidney injury	1 (5.6%)	0 (0.0%)	0 (0.0%)	0 (0.0%)	4 (6.5%)	0 (0.0%)	0.536

### Predictive factors for primary muscle disorder diagnoses

Logistic regression analyses were conducted to identify predictive factors of metabolic myopathy and neuromuscular disease.

For primary disease, the model including male sex and persistent hyperCKemia was significant (Omnibus Test: χ^2^(2) = 30.366, p < 0.001), with a Nagelkerke R^2^ of 0.333 and good model fit (Hosmer–Lemeshow: χ^2^(2) = 1.084, p = 0.582). Both variables were independently associated with diagnosis: male sex (OR = 5.09, 95% CI: 1.792–14.457, p = 0.002) and persistent hyperCKemia (OR = 8.524, 95% CI: 3.145–23.106, p < 0.001).

Male sex remains a predictive factor for both primary disease and metabolic myopathy after excluding X-linked disorders.

### Investigation approach

Initial investigation work-up included a basic metabolic profile in 88 patients (73.9%), performed during acute illness in 33 (37.5%).

Pompe disease screening through acid alpha-glucosidase (GAA) enzyme activity analysis was performed in 39 subjects (32.8%), leading to one diagnosis in this cohort.

Muscle biopsy was performed in less than 10% of patients (n = 11, 9.2%), leading to 7 diagnosis: juvenile dermatomyositis (n = 4), Duchenne/Becker muscular dystrophies (n = 2) and collagen VI-related muscular dystrophy (n = 1). Muscle biopsy was normal in one case and the remaining showed mild, nonspecific myopathic changes, with normal muscle protein staining and no abnormal metabolic features.

Genetic testing was performed in 82 patients (68.9%) including single gene testing for specific gene mutations’ identification and NGS panels for multiple gene analysis.

Single gene testing was carried out in 15 cases (12.6%) and allowed the diagnosis of 6 disorders: 5 cases of McArdle (*PYGM* gene) and 1 case of Pompe disease (*GAA* gene).

Targeted gene panel NGS was performed in 76 (63.9%) patients. Pathogenic or likely pathogenic variants were identified in 16 patients leading to the diagnosis of 11 metabolic myopathies and 4 muscular dystrophies and confirmed the diagnosis of 1 collagen VI-related muscular dystrophy previously identified through muscle biopsy. In one patient, who presented with persistent hyperCKemia, weakness and physical exercise intolerance, NGS identified pathogenic variants in the *TRAPPC11* and *PYGM* genes, leading to the diagnosis of both LGMDR18 and McArdle disease. Overall, the diagnostic yield of NGS analysis was 21.1%. Variants of uncertain significance (VUS) possibly related to disease phenotype were found in 4 patients (5.3%) involving the genes *ENO3* (n = 2), *DMD* (n = 1), and *GIG1* (n = 1). These patients are currently undergoing further investigation. Among the 27 unresolved cases, 9 (33.3%) did not undergo NGS panel testing. However, GAA enzyme activity and single-gene testing for deletions/duplications in the DMD gene were performed in all cases when clinically justified.

The detected mutations and the corresponding genotype–phenotype correlations are detailed in Supplementary Table 2.

**Fig. 4:**
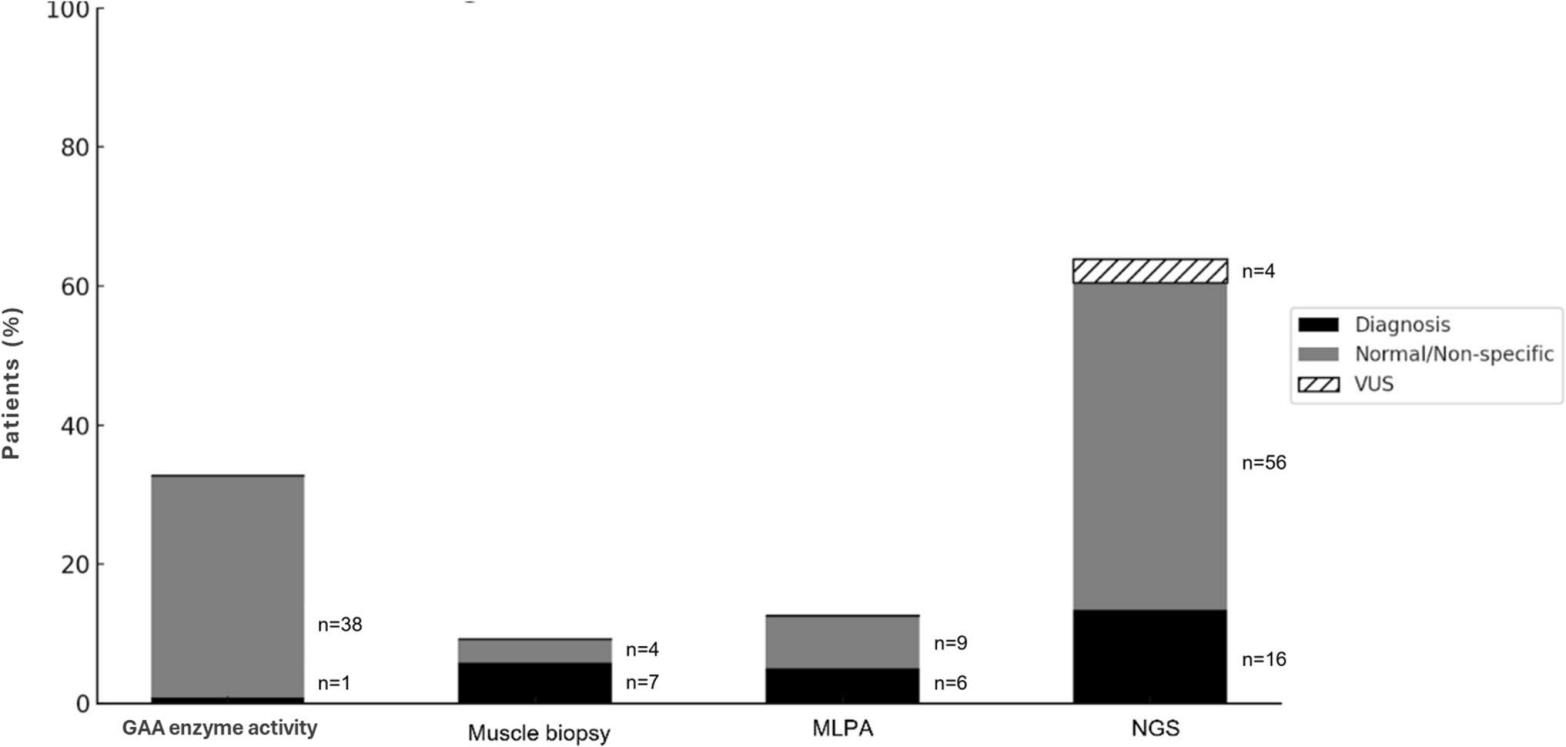
Description of the main investigation methods used in the cohort and respective diagnostic yield

Figure 4 summarizes the main diagnostic methods used.

## Discussion

Clinical presentation remains central to the diagnostic pathway. In our cohort, muscle weakness (48.4%) and exercise intolerance (48.4%) were exclusive to primary cases, reinforcing their diagnostic weight. The second-wind phenomenon was specific for McArdle disease, presenting in 63% of cases. In contrast, secondary causes (mainly viral myositis) were characterized primarily by myalgia and transient CK elevation. These findings echo prior literature indicating that weakness at presentation increases the likelihood of an underlying primary disorder (9).

CK is an important parameter in the assessment of conditions with muscular involvement. Although CK elevations lack diagnostic specificity, certain thresholds and patterns can guide clinical suspicion. In our cohort, most primary disorders occurred in patients with peak CK > 1000 UI/L (83.3%), supporting the use of such cutoffs as an initial stratification tool (17,18). Although peak CK values among primary and secondary cases did not differ significantly in our sample, persistent hyperCKemia emerged as a strong and independent predictor of primary disease. Logistic regression analyses showed that persistence of CK elevation increased the odds of a primary disease eightfold and of metabolic myopathy over 12-fold. Male sex also emerged as an independent predictor in both models, consistent with the known sex distribution of several muscle disorders, and remained significant even after exclusion of X-linked conditions (4). Together, these predictive factors may help clinicians prioritize referral for specialist evaluation and advanced testing.

Our overall diagnostic yield of 25.2% is slightly below the 30% reported in comparable pediatric cohorts (18). Our cohort had a higher proportion of metabolic myopathies (60.0%) probably reflecting an ascertainment bias (patients referred to a Metabolic Diseases Unit rather than a Neurology clinic). However, we had a similar percentage of diagnosis of muscular dystrophies (23.3%).

The diagnostic methodologies applied yielded variable returns. Muscle biopsy, performed selectively in children with suspected muscular dystrophy or inflammatory myopathy, had the highest diagnostic yield (63.6%). This likely reflects appropriate case selection, supporting its role as a second-tier investigation rather than a routine first-line test.

GAA enzyme activity analysis for Pompe disease screening, performed in nearly one third of the cohort (32.8%), yielded a single diagnosis. Although the yield was low, the rationale for including Pompe disease in early testing lies in its “treatable-first” priority: acid alpha-glucosidase deficiency is a progressive disorder with available disease-modifying treatment, where diagnostic delay can worsen outcomes.

Genetic testing provided a diagnostic yield of 26.8%. Single gene testing was diagnostic in 6 of 15 (40%) cases, making it a suitable and cost-effective method when the phenotype is specific. Targeted NGS panels were diagnostic in 17 of 76 cases (22.4%), mostly identifying metabolic myopathies, reinforcing their role in conditions with nonspecific or overlapping presentations with normal or inconclusive laboratory findings. Detection rates using NGS in pediatric populations vary widely in the literature, from 15 to 65% (13,14,19). One study (20) identified a causative genetic variant in 74% of cases using a combination of MLPA, NGS, single-gene testing, and exome sequencing. These differences likely reflect variability in population homogeneity, technological platforms, and analytical strategies. Variants of uncertain significance in 5.3% of our sample highlight ongoing challenges in variant interpretation, especially when testing methods do not fully cover deep intronic variants affecting splicing, large deletions/duplications, or regulatory region changes.

Approximately one quarter of patients (22.7%) in our cohort remain under investigation without a definitive diagnosis; among these, 74.1% presented with a peak CK > 1000 UI/L, and one third (33.3%) with persistent hyperCKemia, suggesting that many may ultimately harbour primary disease. These findings are consistent to another multi-center Portuguese cohort (18). However, interpretation of these results is limited by the fact that, among the 27 unresolved cases, 9 (33.3%) did not undergo NGS panel testing, likely reflecting restricted access to advanced genetic testing during earlier phases of the study period, heterogeneity in diagnostic pathways, and variable clinical suspicion over time.

## Conclusion

Our study highlights the diagnostic challenges and clinical heterogeneity of hyperCKemia in the pediatric population. While many cases are transient and related to benign causes such as viral illness or intense physical activity, a significant subset is attributable to underlying primary disorders, particularly those presenting with persistent CK elevation and symptoms such as muscle weakness and exercise intolerance. Persistent hyperCKemia and male sex emerged as independent predictors of primary disease, highlighting the importance of early risk stratification and targeted evaluation. Genetic testing, especially NGS, showed moderate diagnostic yield and was particularly valuable in identifying metabolic myopathies. A stepwise, phenotype-driven diagnostic approach may be useful to optimize resource use and improve diagnostic accuracy.

## Strengths and limitations

This study included a sizable pediatric cohort with hyperCKemia, providing valuable insights into this understudied population. The rigorous statistical analysis, adherence to clinical guidelines and phenotype-driven approach with clinical features being systematically correlated with diagnostic outcomes are the main strengths of this work. However, it also has some limitations and biases. It was retrospective in nature, and advanced genetic testing was not uniformly available during the earlier phases of data collection. Additionally, there may be a referral bias, as patients were recruited from a tertiary care center, possibly overrepresenting rare disorders. Local referral practices may also have influenced findings with patients with typical features of muscular dystrophy or inflammatory myopathy being likely referred directly to Pediatric Neurology or Pediatric Rheumatology, respectively, bypassing the Inherited Metabolic Disorders Unit. Future prospective studies with standardized protocols and broader recruitment are needed to validate findings and improve diagnostic precision.

## Supplementary Information

Below is the link to the electronic supplementary material.

Supplementary file1 (DOCX 30 KB)

## Data Availability

The data supporting this study are not publicly available due to ethical and privacy restrictions but are available from the corresponding author upon reasonable request and with Ethics Committee approval.
