# Supplementary material for: Pediatric HyperCKemia: a 13-year retrospective study and predictors of neuromuscular disease and metabolic myopathy
Source: Eur J Pediatr. 2026 Apr 10;185(5):250. doi: 10.1007/s00431-026-06914-6 (PMC13068721; doi:10.1007/s00431-026-06914-6)
Supplement: Supplementary file 1 — Supplementary file1 (DOCX 30 KB) [file 431_2026_6914_MOESM1_ESM.docx]

**Table 1A.** Clinical and genetic characteristics of patients with a confirmed primary cause of hyperCKemia, excluding those without genetic testing (n=24).

| **PATIENT NO.** | **AGE** | **SEX** | **PHENOTYPE** | **DIAGNOSIS** |
| --- | --- | --- | --- | --- |
| #1 | 10 | M | Exercise intolerance, muscle weakness | VLCAD (NGS)  *ACADVL*: c.1500_1502delCTT (p.Leu500del) (heterozygous) |
| #2 | 14 | F | Muscle weakness, myalgia | ACAD9 (NGS)  *ACAD9*: c.1552C>T (p.Arg518Cys), exon 11; c.1237G>A (p.Glu413Lys), exon 9 |
| #3 | 17 | F | Exercise intolerance, myalgia, second-wind phenomenon | McArdle (MLPA)  *PYGM*: c.148C>T (p.Arg50*) (homozygous) |
| #4 | 2 | F | Exercise intolerance, myalgia | CPTII (NGS)  *CPTII*: p.Ser113Leu (c.338C>T) (heterozygous) |
| #5 | 18 | M | Exercise intolerance, myalgia, second-wind phenomenon | McArdle (MLPA)  *PYGM*: c.148C>T (p.Arg50*) (homozygous) |
| #6 | 16 | M | Exercise intolerance, myalgia, second-wind phenomenon | McArdle (MLPA)  *PYGM*: c.148C>T (p.Arg50*) (homozygous) |
| #7 | 16 | F | Exercise intolerance, myalgia, second-wind phenomenon | McArdle (MLPA)  *PYGM*: c.613G>A (p.Gly205Ser), exon 5; c.2392T>C (p.Trp798Arg), exon 20 |
| #8 | 1 | M | Incidental | Pompe (MLPA)  *GAA*: c.1082C>T (p.Pro361Leu), exon 7; c.2501_2502delCA (p.Thr834fs), exon 18 |
| #9 | 5 | M | Exercise intolerance, myalgia, second-wind phenomenon | McArdle (MLPA)  *PYGM*: c.148C>T (p.Arg50*) exon 1; c.1827G>A (p.Lys609Lys), exon 15 |
| #10 | 12 | F | Muscle weakness, myalgia | FLAD1 (NGS)  *FLAD1*: c.363G>T (p.Glu121Asp); c.419G>A (p.Arg140His) |
| #11 | 17 | F | Exercise intolerance, myalgia | CPTII (NGS)  *CPT2*: c.887G>A (p.Arg296Gln); c.338C>T (p.Ser113Leu) |
| #12 | 17 | M | Exercise intolerance, myalgia | CPTII (NGS)  *CPT2*: c.338C>T (p.Ser113Leu) (heterozygous) |
| #13 | 5 | M | Exercise intolerance, muscle weakness | McArdle + LGMDR18 (NGS)  *PYGM*: c.1094C>T (p.Ala365Val) (homozygous)  *TRAPPC11*: c.1287+5G>A (r.1114_1287del) (heterozygous) |
| #14 | 6 | F | Muscle weakness | Danon (NGS)  *LAMP2*: exon 1 deletion (heterozygous) |
| #15 | 11 | M | Exercise intolerance, myalgia | McArdle (NGS)  *PYGM*: c.148C>T (p.Arg50*), exon 1; c.2392T>C (p.Trp798R), exon 20 |
| #16 | 17 | F | Myalgia | McArdle (NGS)  *PYGM*: c.148C>T (p.Arg50*), exon 1; c.2392T>C (p.Trp798R), exon 20 |
| #17 | 15 | M | Exercise intolerance; myositis | VLCAD (NGS)  *ACADVL*: c.1500_1502delCTT (p.Leu500del) (heterozygous) |
| #18 | 12 | F | Exercise intolerance; myositis | VLCAD (NGS)  *ACADVL*: c.1500_1502delCTT (p.Leu500del) (heterozygous) |
| #19 | 1 | F | Muscle weakness | LGMD2C (NGS)  *SGCG*: c.848G>A (p.Cys283Tyr) (heterozygous) |
| #20 | 4 | F | Muscle weakness | DMD (NGS)  *DMD*: c.8038C>T (p.Arg2680*) (hemizygous) |
| #21 | 8 | M | Muscle weakness | Dystrophy (NGS)  *DAG1*: c.676C>T (p.Arg226*) (hemizygous) |
| #22 | 2 | M | Muscle weakness | LGMDR18 (NGS)  *TRAPPC11*: c.1287+5G>A (r.1114_1287del) (heterozygous) |
| #23 | 16 | M | Exercise intolerance | Collagen VI-related muscular dystrophy (NGS)  *COL6A3*: c.6130G>A (p.Gly2044Arg) (heterozygous) |
| #24 | 2 | M | Muscle weakness | Dystrophy DMD vs DMB (NGS)  *DMD*: c.3162+2T>A (r.spl?) (hemizygous) |

**Table 1B.** Clinical and genetic features of patients with variants of uncertain significance (VUS) (n=4).

| **PATIENT NO.** | **AGE** | **SEX** | **PHENOTYPE** | **DIAGNOSIS** |
| --- | --- | --- | --- | --- |
| #1 | 15 | M | Incidental | *ENO3*: c.1094dupG (p.Glu366Glyfs) (heterozygous) |
| #2 | 4 | M | Myositis | *DMD*: c.3162+2T>A (hemizygous) |
| #3 | 16 | M | Incidental | *GYG1*: c.304G>C (p.Asp102His) (heterozygous) |
| #4 | 17 | M | Myositis | *ENO3*: c.1099dupG (p.Val367Glyfs*11) (heterozygous) |

**Table 2.** Full list of genes included in the NGS panels.

| ABHD5 | ACAD9 | ACADM | ACADS | ACADVL | ACTA1 | ACTN2 | ACVR1 |
| --- | --- | --- | --- | --- | --- | --- | --- |
| ADSS1 | AGK | AGL | AGRN | ALDOA | ALG13 | ALG14 | ALG2 |
| AMPD1 | ANO5 | ANXA11 | ASCC1 | ASCC3 | ATP2A1 | B3GALNT2 | B3GNT1 |
| B4GAT1 | BAG3 | BET1 | BIN1 | BVES | TWNK | CACNA1S | CAPN3 |
| CASQ1 | CAV3 | CAVIN1 | CCDC78 | CFL2 | CHAT | CHCHD10 | CHD8 |
| CHK8 | CHRNA1 | CHRNB1 | CHRND | CHRNE | CHRNG | CLN3 | CNTN1 |
| COL12A1 | COL25A1 | COL4A1 | COL6A1 | COL6A2 | COL6A3 | COLQ | COQ2 |
| COQ4 | COQ8A | COX6A2 | CTDP1 | CTP2 | CRPPA | CRYAB | DAG1 |
| DES | DGUOK | DHX16 | DMD | DNA2 | DNAJB4 | DNAJB6 | DNM2 |
| DNMT3B | DOK7 | DOLK | DPAGT1 | DPM1 | DPM2 | DPM3 | DTNA |
| DUX4 | DYSF | ECEL1 | EMD | ENO3 | EPG5 | ETFA | ETFB |
| ETFDH | FAM111B | FDX2 | FDX1L | FHL1 | FILIP1 | FKBP14 | FKRP |
| FKTN | FLAD1 | FLNC | FXR1 | GAA | GBE1 | GFER | GFPT1 |
| GGPS1 | GMPPB | GNE | GOLGA2 | GOSR2 | GYG1 | GYS1 | HADHA |
| HADHB | HACD1 | HMGCR | HNRNPA1 | HNRNPA2B1 | HNRNPDL | HRAS | HSPB1 |
| HSPB8 | HSPG2 | INPP5K | ISCU | ISPD | ITGA7 | JAG2 | KBTBD13 |
| KCNJ18 | KLHL40 | KLHL41 | KLHL7 | KLHL9 | KY | LAMA2 | LAMA5 |
| LAMB2 | LAMP2 | LARGE1 | LDB3 | LDHA | LIMS2 | LMNA | LMOD3 |
| LPIN1 | LRIF1 | LRP4 | MAP3K20 | MATR3 | MB | MCOLN1 | MEGF10 |
| MICU1 | MLIP | MPDU1 | MSTN | MSTO1 | MTM1 | MTMR14 | MUSK |
| MYBPC1 | MYBPC3 | MYH2 | MYH3 | MYH7 | MYL1 | MYL2 | MYMK |
| MYO18B | MYO9A | MYOD1 | MYOT | MYPN | NEB | NEFL | OBSCN |
| ORAI1 | PABPN1 | PAX7 | PIP5K1C | PFKM | PGAM2 | PGK1 | PGM1 |
| PHKA1 | PHKB | PIEZO2 | PLEC | PLIN4 | PNPLA2 | PNPLA8 | POLGUT1 |
| POLG | POLG2 | POMGNT1 | POMGNT2 | POMK | POMT1 | POMT2 | POPDC3 |
| PPA2 | PREPL | PRKAG2 | PTPLA | PTRF | PUS1 | PYGM | PYROXD1 |
| RAPSN | RBCK1 | RPH3A | RRM2B | RXYLT1 | RYR1 | RYR3 | SCN4A |
| SELENON | SEPN1 | SGCA | SGCB | SGCD | SGCG | SIL1 | SLC16A1 |
| SLC18A3 | SLC22A5 | SLC25A1 | SLC25A20 | SLC25A4 | SLC5A7 | SMCHD1 | SMPX |
| SNAP25 | SPEG | SPTBN4 | SQSTM1 | STAC3 | STIM1 | SUCLA2 | SVIL |
| SYNE1 | SYNE2 | SYT2 | TAFAZZIN | TANGO2 | TAZ | TCAP | TIA1 |
| TK2 | TMEM43 | TMEM5 | TNNC2 | TNNI2 | TNNT1 | TNNT3 | TNPO3 |
| TOR1AIP1 | TPM2 | TPM3 | TRAPPC11 | TRDN | TRIM32 | TRIM54 | TRIM63 |
| TRIP4 | TSEN54 | TSFM | TTN | TWNK | TYMP | UNC13A | UNC45B |
| VAMP1 | VCP | VMA21 | VPS33B | VWA1 | XK | YARS2 | ZC4H2 |
